# Note on the Reliability of Biological vs. Artificial Neural Networks

**DOI:** 10.3389/fphys.2021.637389

**Published:** 2021-02-12

**Authors:** Ruedi Stoop

**Affiliations:** ^1^Department of Physics, Institute of Neuroinformatics, University and ETH Zürich, Zurich, Switzerland; ^2^School of Electronic Information Engineering, Technical University of Xi'an, Xi'an, China

**Keywords:** artificial vs. biological neural networks, size and structure of problem solutions, network solution reliability, generalization ability, influence of evolution

## Abstract

Various types of neural networks are currently widely used in diverse technical applications, not least because neural networks are known to be able to “generalize.” The latter property raises expectations that they should be able to handle unexpected situations with similar success than humans. Using fundamental examples, we show that in situations for which they have not been trained, artificial approaches tend to run into substantial problems, which highlights a deficit in comparisons to human abilities. For this problem–which seems to have obtained little attention so far–we provide a first analysis, based on simple examples, which exhibits some key features responsible for the difference between human and artificial intelligence.

## Introduction

The evolution of the species and how it works remains a domain of vibrant discussion; for a recent overview see, e.g., Smith (2011). As one illustrative example, the hearing of mammalians has converged toward different variants of the cochlea all based on the same architectural prototype (Lorimer et al., 2015), whereas for reptiles a similar convergence is not observed. Generally, the evolution of physiological networks has led to ever more dedicated structures that are able to respond in an ever more efficient way to the challenges posed by the context in which life happens (Hofman, 1985), whereas for no obvious reasons, evolution has ignored the potential for more optimal implementations in other cases. Despite the fact that it is not fully understood how evolution brings about features of intelligence, mimicking key features of it has proven to be a powerful tool for obtaining excellent technical solutions in a wide range of fields (Rechenberg, 1973). However, our partial insight into how biological evolution works must be expected to have hindered the full unfolding of the power of this method. Conversely, the application of accepted key principles of evolution in the search of technical solutions may also provide an indirect test on how well we actually understand biological evolution.

One puzzling issue raised by the solutions obtained in this way is that they are often much larger than what we would expect from a comparison with corresponding specialized biological networks (cf. Lorimer et al., 2015). Would smaller solutions not generally be the better solutions, and if so, why do some biological solutions not care much about this? Moreover, why do both biological and artificial solutions emerge for which we are unable to extract the underlying logical structures? Critical questions of this kind are often generously waived by referring to the presently available large number of computational elements in computers, and to the human cortex's incredible number of neurons. In the following investigations we argue that such arguments fall short of rendering justice to the issue.

## Evolution Toward Optimal Solutions in Neural Networks

A first insight may be gained by looking at how the evolutionary algorithm paradigm works on classical artificial neural networks. As both a benefit and a disadvantage at the same time, any high-dimensional function can be represented by a sufficiently large feed-forward neural network (Cybenko, 1989; Hornik, 1991; Lu et al., 2017). Traditional feed-forward neural networks implement functions by taking input and converting it, passing through a network which often has several layers, into output that normally triggers some follow-up action. One particularity of neural networks is that the information flow of the system (hand-designed or found by genetic programming, see later) is not required to be given. Rather, the information flow emerges through a learning process that in a sufficiently large network arranges the connection weights between the computational elements in such a way that the desired results, or actions, are triggered.

With the desired reactions as the network's goal function, learning by gradient-descent optimization on the weights toward their optimal values creates decision hypersurfaces. Unfortunately, the cases where the optimal decision boundaries alone may not be unique, the description of a given decision boundary in a space of network weighs much more than ultimately necessary, cannot be expected to be unique. Instead, the description will depend on initial conditions, on the network's elements used, and on the sequence of learning inputs, as, e.g., distinct weight combinations may implement the same geometric object. Because small weights may lead to computational problems in the gradient descent, convolutional layers in the networks often provide a substantial improvement, if the nature of the relevant filters to be implemented is known (Krizhevsky et al., 2017). However, this “deep layer network approach” may be seen to suffer from most of the described shortcomings just the same.

We first show, using simple examples, that optimization based on the main principles of evolution, may in principle provide the simplest solution structure for neural network solutions. In our example, we use the method of evolutionary optimization on a population of classical one-hidden-layer neural networks of a fixed more-than-sufficient size. We apply a fitness function *F*_λ_ = *Q*(*G*) − λσ(*G*) combining the unconstrained fitness function *Q*(*G*) of the network *G* with a cost term constraint σ(*G*) that measures the size of the network *G*. λ ∈ [0, 1] is a parameter that, upon its increase, may be biased from essentially fully connected (λ = 0) networks that solve the desired task, to the sparsest networks with the latter property (a similar cost term can alternatively or additionally be applied directly on the learning).

Heading for a sufficiently simple task, we search for neural networks that implement logical gates, in our case AND XOR, in the most efficient way (universal gates could be treated similarly). For our simulations, we started from a population of 20 fully connected networks with two inputs implementing the two logical input channels, connecting to one hidden layer of 30 neurons, followed by a layer of one output neuron providing the result of the desired logical function.

**Figure 1:**
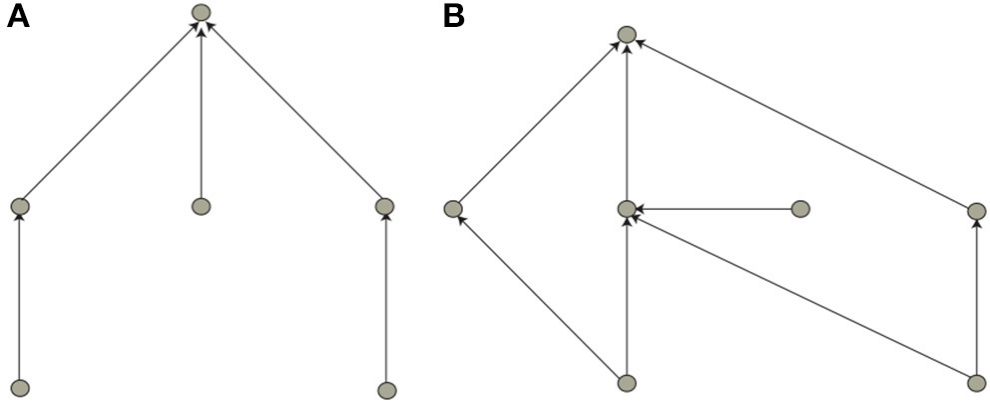
Minimal solutions for the neural network implementing logical functions, **(A)** AND and **(B)** XOR. Input (e.g., a picture via its pixels) is fed into the network from the bottom (into the input layer), processed higher up in the so-called hidden layer, and the result of this computation is fed into the output layer. Due to the simplicity of the binary logic problem, one single output neuron is sufficient for the latter. In our approach, based on neurons with zero firing threshold, hanging inputs (i.e., neurons not receiving input from lower-level network layers) denote a “bias” connection implementing a firing threshold.

After each period of learning with fixed network architecture guaranteeing each network to converge onto its best possible solution behavior, the evolutionary process was applied, using parental choice of networks by a wheel of fortune involving the fitness *F* of the population members. For the next generation, parent networks are cut into two parts each and recomposed toward the new generation. Eventually, mutation was applied, and very small weights were set to zero. Running this standard evolutionary paradigm (Steeb, 2015) for a sufficient number of generations, yields perfect implementations of the desired logical blocks, see Figure 1, for mild values of the parameter λ.

Our experiments corroborate that by using fitness functions *Q*(*G*) with a cost term constraint σ(*G*) punishing for the size of the network, after a sufficiently long evolution, the minimal logical structure of a problem's solution can be obtained. While this may be highly desirable, in particular from various theoretical points of view, the question of how much this bears relevance for practical applications remains.

## Genetic Programming Approach

To investigate this issue, we turn to problem solutions generated using the genetic programming paradigm (Koza et al., 1999). While various variants of genetic programming have been developed in the recent past (differing somewhat in ease and elegance of implementation), there are no generally accepted distinctions regarding the quality of the obtained solutions (in particular not for the aspect that we will be interested in) (Oltean and Grosan, 2003). For a least biased ansatz, we do not revert to the most sophisticated (e.g., gene expression programming, Ferreira, 2001) versions of the approach. We content ourselves with using a simple evolutionary optimization on a population of random programs composed of fundamental logical operations and constants. Specifically, our task will be that of providing a robot with a program to follow the walls in a grid of two-dimensional arrays (Zhu, 2003). Sensors are used to detect in space-fixed “n” (north), “ne” (northeast), “e” (east), “se” (southeast), “s” (south), “sw” (southwest), “w” (west), “nw” (northwest) directions, whether neighboring space sections are occupied by the wall. Based on this input, the robot's program then determines the next horizontal or vertical one-step move (into the “north,” “east,” “south,” “west” direction, respectively). For the composition of a candidate program, primitive functions IF, AND, OR, NOT, and the constants “True” and “False” are available; they allow for the modeling of any desired program (alternatively, universal logical building blocks NAND, or NOR could be used). An optimal program is found if after hitting a wall, the wall is followed, no matter what the initial condition would be (Zhu, 2003). Using the evolutionary approach, the optimal program can be found as follows. A population of random programs is created by randomly connecting a selection of the mentioned elements, with, for convenience, a biased selection of the syntactical elements IF, AND, OR, NOT. Programs are fitness-rated according to how many times different parts of the wall are visited during a journey of sufficient but fixed length, starting from a number of random initial conditions. A wheel of fortune chooses parent programs that through crossing and mutation create new, often suboptimal, programs, but bend the population in the usual manner of evolutionary algorithms that foster the exploration of a generally multi-facetted, multi-rigged fitness landscape, toward more optimal solutions.

Running this evolutionary paradigm terminates after a search of many evolutionary steps with distinct optimal programs (i.e., programs that perform the desired task in a perfect way, starting from arbitrary initial positions), such as (we use Mathematica notation that abbreviates AND, OR, and NOT by, &&, ∣∣, and !, respectively):

Sol1=(!If[*s*, If[!(!nw&&If[se, !west, north]), north,

If[!(*n*||If[If[!west, ne, !If[west, se, nw]], nw, south&&!(*w*&&*n*)]||(west&&If[*w*, east, If[If[*s*, If[!west, !If[west, se, nw], *n*],

If[ne, south, east]], If[!*n*||east, north, (north&&north)||sw],

If[!If[north, *n*, If[west,

(!If[*s*, If[west, !If[ne, !sw, west], east],

If[ne, south, nw]]&&west&&south)&&If[se, !west, south], *n*]]&&south, sw, True]]])||se),

!If[south, se, nw], *n*]], If[If[*s*, west, If[se, south, If[!*n*||east, north,

!(!(east&&nw)&&south&&east)]]], south, east]]&&south&&*e*),

||south

or

Sol2 = If[If[!If[*e*, If[If[*e*, nw, ne], *w*, north]||*s*, If[se, east, False]],

If[*w*, nw&&!south, *w*], *s*], west&&*n*, west]||(east&&!*w*)

If[If[!If[*e*, If[If[*e*, nw, ne], *w*, north]||*s*, If[se, east, False]],

If[*w*, nw&&!south, *w*], *s*], west&&*n*, west]||(east&&!*w*),

or

Sol3 = If[If[*e*, If[sw, west, se]&&south, !If[!ne&&*w*, ne||north,

If[!If[ne, east, *w*], *n*, east]&&east]]&&west, south, east]

If[If[*e*, If[sw, west, se]&&south, !If[!ne&&*w*, ne||north,

If[!If[ne, east, *w*], *n*, east]&&east]]&&west, south, east].

**Figure 2:**
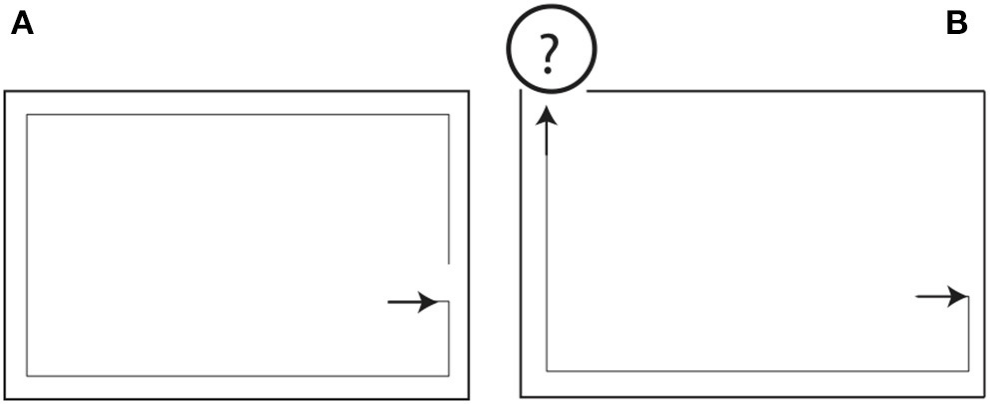
Perfect solutions for the wall-following task from an initial population of random programs as solution candidates, in the case of **(A)** a closed, **(B)** an open labyrinth. How the robot will perform under new conditions outside the labyrinth, is unclear (see text).

While all of these programs solve the posed problem in a satisfactory manner (i.e., they guide the robot from an arbitrary starting point straight to the wall whereupon the wall is followed in the desired manner, see Figure 2A for an illustration), the reader will remark that even under application of substantial efforts, these programs are not understandable (translation into alternative notations does not remedy this).

## Non-Equivalence of Solutions

Even if, depending on a reader's background, champion programs from a certain variant of the genetic programming approach may appear to be more readable than those from another approach, they all provide only limited insight into how they actually work (Paterson, 2002; Ortega et al., 2007). Naturally, the question arises whether all of these programs are not just equivalent formulations (e.g., modulo simplification using de Morgan's laws^1^), or whether they are, in some sense, indeed different.

To answer this question, one can design a test function *T* that for a found solution will evaluate the result (“True” or “False”) on all possible (in our case 2^12^) system states. Comparing the last two solutions, we observe that out of 4,096 possible cases, the solutions differ on 1,216 cases, and an even bigger disagreement can be observed with respect to the first and the latter two solutions (disagreement in more than 2,000 cases). This result points to a substantial problem involved in this paradigm of solution construction. It means that the robot's behavior outside the labyrinth (cf. Figure 2B), when facing challenges distinct from those experienced within the labyrinth, is essentially unpredictable.

## What Is in Larger Biological vs. Smaller Artificial Solutions?

Why then does biology not simply implement the simplest (smallest, simplest-to-read) solutions? The answer has to do with the structurally stable solutions that emerge from evolution (at a level that disregards the variability of solutions required by evolvability as the underlying principle of evolution over shorter time-scales). To arrive at the simplest solution, our problems were presented over and over, with no variation. This, however, is not even close to how evolution takes place in the real world. Real-world evolution is known to take place in rigged, ever-changing fitness landscapes, leaving in a species' DNA or, more generally in a “solution,” elements of ancient experience of the interaction with it. The implication that we may draw from this is: the smaller its size and therefore also the clearer its logical structure is, the more ignorant a network will be regarding other issues that it might, occasionally, be confronted with. Reversing the above argument, to be prepared for unexpected challenges, a network thus must be larger than minimally necessary for solving a task, even if it will be engaged mainly in solving this task in the most modest context. One distinction of artificial vs. animal intelligence is thus that in both artificial approaches that we have taken above, we have not included the physical reality that a real-world decision system has been confronted with, and has been shaped by, during evolution. Physical reality is not an arbitrary structure but reflects an “inner connectivity” of the world around us; fitness landscapes do not change in an arbitrary manner, but their changes are constrained to respect the environment's deeper construction rules, such as the laws of physics or chemistry that reflect it. Changes in the fitness landscape occur on time-scales similar to those required in terms of generations for population optimization (e.g., ice ages). Therefore, they must be expected to leave long-term traces in the genetic code, providing to a real-world system guidelines for the encounter with “unexpected” situations that are better than arbitrary ones. For technical applications, the inclusion of corresponding properties into the search of optimal solutions may be feasible and may lead to solutions with an improved robustness for the encounter with unexpected situations.

In a reformulation of our insights for the biological genetic context, we might say that animal (and in particular: human) intelligence seems to be founded on an implementation method similar to the one used by the biological DNA: DNA cannot define every cell of an individual individually but is sufficient to act as the key to a factory offered by the environment (a mother's womb, or more generally physical and social conditions). Together, through their interaction they produce the final living system, similar to how initial conditions lead dynamical systems to a final solution. Such an interpretation would be consistent with the observation that the more controlled the environment of a living system is, the smaller its amount of DNA generally is [e.g., the human DNA is, measured in base pairs (~ 3 · 10^9^ base pairs). Much smaller than that of a certain very ancient lungfish protopterus aethiopicus (~ 140 · 10^9^), or of a certain amoeba dubia (~ 670 · 10^9^), or even that of an onion (~ 18 · 10^9^), lilies (~ 90 · 10^9^) or that of Pinus, cf. Ideker et al., Gregory, 2000, 2001; Morse et al., 2009]. The size of the brain, in contrast, can be seen as a mapping of the reality as perceived by a species, making species with a larger brain more successful in dealing with the reality they are embedded in. Finally, it is clear that the societal structures that living systems are embedded in, may also leave traces, beyond long term societal beliefs or memories (the latter manifesting themselves perhaps, e.g., in mythological pictures like dragons). However, this influence not only appears to be much weaker, but will also be more difficult to assess, due to its nature. Studying phenomena like the internet from this perspective might, however, shed light on the modalities of this influence.

## Data Availability Statement

The raw data supporting the conclusions of this article will be made available by the authors, without undue reservation.

## Author Contributions

The author confirms being the sole contributor of this work and has approved it for publication.

## Conflict of Interest

The author declares that the research was conducted in the absence of any commercial or financial relationships that could be construed as a potential conflict of interest.

## Footnotes

^1^We use, e.g., in logic, circuitry, or set theory, de Morgan (and other, like double negation) rules to simplify expressions. An example would be ¬(*p* ∨ (¬*p* ∧ *q*)) ∨ (*p* ∨ *q*) ≡ (¬*p* ∧ ¬*q*) ∨ (*p* ∨ *q*) ≡ ¬(*p* ∨ *q*) ∨ (*p* ∨ *q*) = always true (tautology).
